# Supplementary material for: Elucidating the causal mechanisms of Taenia solium transmission in humans, pigs and the environment: A global systematic review
Source: PLoS Negl Trop Dis. 2026 May 12;20(5):e0014281. doi: 10.1371/journal.pntd.0014281 (PMC13183288; doi:10.1371/journal.pntd.0014281)
Supplement: S1 File — (DOCX) [file pntd.0014281.s001.docx]

**File S1: Modifications made to the Quality Assessment Tool for Quantitative Studies**

This document describes the original Quality Assessment Tool for Quantitative Studies, our modifications to it, and our justifications for those modifications. Our modified form, with quality ratings for each data element, is included in File S2.

**Part A: Selection Bias**

**Question 1** (original): Are the individuals selected to participate in the study likely to be representative of the target population?

1. **Very likely**
2. **Somewhat likely**
3. **Not likely**
4. **Can’t tell**

**Modifications**: As anyone could become exposed to the eggs of *Taenia solium*, everyone is a part of the population modeled within the DAG. Additionally, although the original data dictionary included patient selection method in this question, we dropped this part from our interpretation. Many studies with volunteers had high levels of participation (>80%). At those levels, the risk of volunteer bias is low, and lower levels of participation would penalize this category’s rating through Question 2 anyway.

Interpretations of each level are as follows:

1. **Very likely** – No exclusion criteria were used by the study
2. **Somewhat likely** – The study used exclusion criteria, but the criteria were so narrow that the study results are likely still applicable to the target population. The most common example of such a criterion is excluding children ≤2-5 years old. Studies that exclude pigs ≤6 months old are included here because, although pigs reach sexual maturity at 5-6 months, they continue growing until they are 18 months old.
3. **Not likely** – The study used broad exclusion criteria or had a population so severely skewed demographically that the applicability of the study to the target population is limited. Any study that excludes the majority of an at-risk group (children, adults, males, females etc.) or that selects only those in one demographic group (occupation, location requirement beyond endemicity of the parasite) should be rated as “not likely”. For pigs, populations that come from slaughter surveys are not representative because pigs sent to slaughter are likely adults.
4. **Can’t tell** – This was not assigned to any studies in our analysis. As our studies had to include information about a potential association (of the form exposure leading to outcome), all studies had to include at least some information on the sampled population. Thus, information in the study was sufficient to assign studies to one of the three levels above.

**Question 2** (original): What percentage of selected individuals agreed to participate?

1. **80-100% agreement**
2. **60-79% agreement**
3. **<60% agreement**
4. **Not applicable**
5. **Can’t tell**

**Modifications**: If the sample was selected systematically, then this question referred to the percentage of those approached who agreed to participate. If the sample was composed of volunteers, then this question referred to the percentage of the eligible population who agreed to participate.

Interpretations of each level are as follows:

1. **80-100%** - Same
2. **60-79%** - Same
3. **<60%** - Same
4. **Not applicable** – All of our studies had a sample that was systematically chosen or chosen at random. Thus, this category was not used.
5. **Can’t tell/ Implied 100%** - This was used for studies that did not report the total numbers of those approached (if systematic) or those eligible (if volunteer).

**Additional modifications:** Generalizability of the study populations and percentage of recruitment are not the only determinants of selection bias. Thus, we created the following questions and added them to our measure:

1. **Exposure**: Independent variable measured by the study
2. **Possibility of bias with regards to the exposure**: Whether study selection could have altered the distribution of the exposure in the study sample. Possible answers were
   1. **No** – Non-random bias due to selection is not possible
   2. **Can’t tell** – The study had insufficient information to determine whether study selection created a bias on the distribution of the exposure
   3. **Indirect possible** – A specific aspect of study selection likely acted on a cause of the exposure
   4. **Direct possible** – A specific aspect of study selection may have acted directly on the exposure
   5. **Direct known** – A specific aspect of study selection clearly affected the distribution of the exposure
3. **Outcome**: Dependent variable measured by the study
4. **Possibility of bias with regards to the outcome**: Whether study selection could have altered the distribution of the outcome in the study sample. Possible answers were
   1. **No** – Non-random bias due to selection is not possible
   2. **Can’t tell** – The study had insufficient information to determine whether study selection created a bias on the distribution of the outcome
   3. **Indirect possible** – A specific aspect of study selection likely acted on a cause of the outcome
   4. **Direct possible** – A specific aspect of study selection may have acted directly on the outcome
   5. **Direct known** – A specific aspect of study selection clearly affected the distribution of the outcome
5. **Selection bias possible presence** – By considering the results of questions 2 and 4, is there likely selection bias in this study? Possible answers were
   1. **No** – Question 2 is “no” OR Question 4 is “no”
   2. **Yes** – Questions 2 AND 4 answered with any other answer than “no”
6. **Rationale**: A write-in question for reviewers to explain their reasoning for Question 5. If Question 5 was answered with “yes”, then reviewers would also write in their predictions at the effects of the selection bias.

While reviewers discussed disagreement between answers on these additional questions, only Additional Question 5 was considered for disagreement.

**Rating** (original):

1. **Strong**: Q1 is “very likely” AND Q2 is “80-100%” AND Additional Question 5 is “no”
2. **Moderate**: Q1 is at least “somewhat likely” AND Additional Question 5 is “no”
3. **Weak**: Q1 is “not likely” OR Additional Question 5 is “yes”

**Modifications**: None

**Part B: Study design**

**Question 1** (original): Indicate the study design

1. **Randomized controlled trial**
2. **Controlled clinical trial**
3. **Cohort analytic (two-group pre + post)**
4. **Case-control**
5. **Cohort (one group pre + post)**
6. **Interrupted series**
7. **Other (specify)**
8. **Can’t tell**

**Modifications**: The two cohort types were condensed into the single category of “Cohort”. Three additional types of studies (“Qualitative”, “Descriptive”, and “Cross-sectional”) were included in this list. “Other (specify)” was removed.

**Questions 2, 3, and 4** (original): These questions ask whether the study was randomized, whether the randomization method was described, and whether the randomization method was appropriate.

**Modifications**: Only one randomized controlled trial was identified in our search. Since there was low demand, we eliminated these questions in order to shorten the quality form.

**Rating** (original)

1. **Strong** – The study is a controlled trial (randomized or clinical trial)
2. **Moderate** – The study uses a cohort, case-control, or interrupted series method
3. **Weak** – The study used another method or the method could not be identified

**Modifications**: Cross-sectional studies are rated as “Moderate”. “Qualitative” and “Descriptive” studies are rated as “Weak”. No modifications were made to the ratings of other study types.

**Part C – Confounders**

This section assesses the presence of confounders in the study and the percentage of those that were controlled for. In our form, confounding caused by differences between the sample population and the target population are assessed in Part A. As for controlling, whenever possible, we sought unadjusted results so that we could have a common comparison when judging the strength of this connection. Thus, this part of the tool was **removed** from our analysis.

**Part D – Blinding**

This section assessed whether the evaluators and participants were blinded as to the exposure status (evaluators) or to the research question (participants). Since the majority of our identified studies were cross-sectional, exposure and outcome status were determined at the same time. Thus, this part of the tool was **removed** from our analysis.

**Part E – Data Collection Methods**

**Question 1** (original): Were data collection tools shown to be valid?

1. **Yes**
2. **No**
3. **Can’t tell**

**Question 2** (original): Were data collection tools shown to be reliable?

1. **Yes**
2. **No**
3. **Can’t tell**

**Grading** (original)

1. **Strong** – the tool is valid (Question 1 is “Yes”) and reliable (Question 2 is “Yes)
2. **Moderate** – the tool is valid (Question 1 is “Yes) but not reliable (Question 2 is “No” or “Can’t tell”)
3. **Weak** – the tool is not valid (Question 1 is “No” or “Can’t tell”)

**Modifications**: This part was completed twice for each data element. The first was completed for the tool that measured the exposure of the potential association and the second was completed for the tool that measured the outcome. See the Excel file (File S2) for what is considered valid and reliable for each exposure and outcome.

**Part F – Withdrawals and Drop-outs**

This section assesses whether study integrity was affected by people leaving over the course of the study. As the majority of our identified studies were cross-sectional, this section was **removed** from our analysis.

**Part G – Intervention Integrity**

This section assesses whether participants received the intervention equally. Although the first question (about the percentage of participants who receive the intervention) could also apply to the exposure, this part as a whole is more important to interventional studies. Since our studies were about exposures instead of interventions, this section was **removed** from our analysis.

**Part H – Analyses**

**Question 1** (Original): Indicate the unit of allocation

1. **Community**
2. **Organization/Institution**
3. **Practice/Office**
4. **Individual**

**Modifications**: This question referred to the unit of exposure rather than the unit of allocation. The level of exposure is determined by the question asked. Thus, a household- or community-level study (for example, where one individual is studied per household) may measure an individual-level outcome (for example, gender or age) while an individual-level study (where multiple individuals were sampled per household or where individuals were sampled without regard to the number in the household) may measure a household- or community-level factor (for example, whether the household has a latrine). Finally, since we excluded studies that recruited ill patients from hospitals, this level was dropped. The levels were as followed:

1. **Community**
2. **Household/concession**
3. **Individual**
4. **Can’t tell**

**Question 2**: Indicate the unit of analysis

1. **Community**
2. **Organization/Institution**
3. **Practice/Office**
4. **Individual**

**Modifications**: The possible units of analysis were changed to match the possible units in Question 1 (possible exposure units). The analysis level is dependent on both the sampling method and the exposure of interest. If data were collected at the individual level (where multiple individuals were sampled per household or where individuals were sampled without regard to the number in the household), and if no group outcome information is presented, then the analysis MUST be individual because we cannot calculate a group measure on our own. If data were collected at a household or a community level (for example, where one individual is studied per household) and the unit of exposure is an individual, then the unit of analysis is also individual because data can be analyzed at the individual level. However, if the unit of exposure is household, then the unit of analysis is also household if only one individual was examined per household.

**Question 3**: This question asked whether the statistical methods were appropriate for the study design. Since we performed our own statistics in many cases, this question was **removed** from our analysis.

**Question 4**: This question asked whether an intention-to-treat analysis was used. Since the vast majority of our identified studies are not identified, this question was **removed** from our analysis.

**Grading**: The tool does not describe the grading for this section

**Modifications**: A new question was added to this section. If the units of exposure and the units of analysis do not match, then the results may be have artificially decreased variance. Thus, if we do not know that the units of exposure and the units of analysis match, the quality of the paper is weaker than it would be if the units matched. We did not account for whether the authors adjusted for clustering directly in this quality review because, whenever possible, we extracted crude (unadjusted) results to ensure our selected data was not preemptively adjusted for confounding by other potential risk factors.

**Question** (new): Does the unit of exposure and the unit of analysis match?

1. **Yes** – Equivalent to a “Moderate” or “Strong” rating as used in other sections
2. **No** – Equivalent to a “Weak” rating as used in other sections
3. **Can’t tell** – Equivalent to a “Weak” rating as used in other sections.

**Global Rating**

The original ratings are

1. **Strong** – No “Weak” ratings
2. **Moderate** – One “Weak” rating
3. **Weak** – Two or more “Weak” ratings.

We did not make any modifications to the global rating process.
