# Supplementary material for: Elucidating the causal mechanisms of Taenia solium transmission in humans, pigs and the environment: A global systematic review
Source: PLoS Negl Trop Dis. 2026 May 12;20(5):e0014281. doi: 10.1371/journal.pntd.0014281 (PMC13183288; doi:10.1371/journal.pntd.0014281)
Supplement: Attachment — Submitted filename: Reviewer comments_ PNTD-D-25-00379.docx [file pntd.0014281.s006.docx]

**Reviewer comments**

Thank you for the opportunity to review this important piece of work. Overall, this is a well thought research process which addresses a unique gap in evidence. The work will be very valuable to people wishing to set up studies looking at risk factors of the different *T. solium* diseases. The work deserves to be published after addressing the comments raised below.

**Methodology:** The authors need to provide a justification on why they had to use other options to obtain so much articles (694). Ideally the search syntax developed should be able to return most of the relevant information. Additional search may be done but mostly for grey literature. The SLR process should be rigorous and replicable. It is not clear the process followed to obtain the additional 694 articles and is it replicable? This an important flaw and needs to be clarified or the approach explained and justified.

The search syntax used seemed to have been very general and may have resulted to so many irrelevant hits.

**Results:** The results on the quality appraisal of the studies have not been reported.

**Discussion:** I find the discussion section shallow and lacking enough details to explain some of the important findings reported in this study. For examples the authors should discuss the variation in the strength of evidence as reported by the authors,- is it due to issues to do with how the study was conducted, sample size or other contextual issues. A discussion on what was used to gauge the strength of evidence – was it p values only or were Confidence intervals also considered? What creates the differences in the number of studies conducted across the different geographical regions. The design of the studies needs to be discussed especially for those focusing on HCC as all this can have an influence on the results reported in those studies.

Other comments are as outline below:-

Line 9 : - consider rewording that sentence, the individual diseases in themselves are not complex rather the entire value chain of the parasite.

Line 49: the word “lays” sound abit off, may be consider using “produce” instead.

Line 50 – 51 does not read very well , may be it should “…the eggs which develop to larval cysticerci….”

Line 63 to 64: What does the word “human control” mean please make the sentence clear and complete.

Line 78: you should use past sentence throughout the write up.

Figure 4: make the colours clear and distinct , currently the blue is light blue and what is referred to as red is brown. The labelling should also be improved so that the words are clear and readable so that some letters don’t appear to be sitting on top of others. SES should be defined.

Figure 5: see comment above on colours. I cannot find colour orange and grey

Line 282 to 283: see comment on the need to discuss some of these observations. In some areas the uncastrated boar is used for mating across villages and therefore we expect the pig to have more chances of picking infective eggs as it moves from one farm to the other were management practices may vary.

Line 297: Women keep only sheep or sheep and goats – probably use small ruminants to accommodate variations across regions

Line 331 to 334: This may not be a correct observation- see findings in (<https://journals.plos.org/plosntds/article?id=10.1371/journal.pntd.0003570>) and (<https://www.frontiersin.org/journals/veterinary-science/articles/10.3389/fvets.2022.833721/full>).

Line 354: what does that sentence mean, clarify or delete.
