# Supplementary material for: Elucidating the causal mechanisms of Taenia solium transmission in humans, pigs and the environment: A global systematic review
Source: PLoS Negl Trop Dis. 2026 May 12;20(5):e0014281. doi: 10.1371/journal.pntd.0014281 (PMC13183288; doi:10.1371/journal.pntd.0014281)
Supplement: Attachment — Submitted filename: response letter 20260124 clean.docx [file pntd.0014281.s007.docx]

Dear PLOS Neglected Tropical Diseases editors,

Thank you very much for sending us your decision on our initial submission of our article, “Elucidating the causal mechanisms of *Taenia solium* transmission in humans, pigs and the environment: a global systematic review.” We greatly appreciate the feedback from the reviewers, and we feel that addressing their comments has improved the quality of our manuscript. We have included a line-by-line response to the **reviewers’ comments and suggestions** below.

We also appreciate your responsiveness to our questions during the revision process. We have altered our manuscript following your requirements.

**Addressing Reviewer 1’s Comments**

**Thank you for the opportunity to review this important piece of work. Overall, this is a well thought research process which addresses a unique gap in evidence. The work will be very valuable to people wishing to set up studies looking at risk factors of the different *T. solium* diseases. The work deserves to be published after addressing the comments raised below.**

Thank you for your kind words and thorough review.

- **The authors need to provide a justification on why they had to use other options to obtain so much articles (694)… The SLR process should be rigorous and replicable. It is not clear the process followed to obtain the additional 694 articles and is it replicable?**

Thank you for this important comment. While the terms included in our search phrase for the bibliographic database search were general, the use of “epidemiology” may have excluded relevant studies that explored associations among risk factors and the TSCT outcome without mentioning the “epidemiology” in their publication. In hindsight, this should have been corrected, but changing the strategy now would mean redoing the whole review, which is not realistic at this time. However, we believe that we were able to capture most relevant publications through screening papers cited in previous literature reviews. This allowed us to identify 136 additional articles eligible for full-text review, of which 21 were included in the review, representing 13% of all included articles, but only 3.5% of articles screened that way. In contrast, of the 1146 articles identified through databases or registers that were screened, 11% were included. Although this does not guarantee that the approach was perfect, it suggests that most of the available literature was probably captured. All articles included in the reference lists of narrative or systematic reviews identified during the bibliographic database search underwent the same systematic review process (ie duplicate removal, title abstract and full text screening). We therefore feel that this part of the review process is also truly systematic and reproducible.

We have now added a paragraph in the Methods section on page 8 describing the procedures related to the screening process for the reference lists of the systematic review articles. We have also added a limitation to our search strategy in the discussion section.

*A second limitation of our review is that we might not have captured all relevant literature in the field. First, the use of the term “epidemiology” in our search phrase may have excluded relevant studies that explored associations among risk factors and the TSCT outcome without mentioning the term “epidemiology” in their publication. However, we feel we have largely compensated for this by screening all articles cited in systematic reviews identified through our database search. Second, because we restricted inclusion to peer-reviewed journal articles, we may have missed relevant evidence from the grey literature (e.g. reports, theses, conference proceedings), potentially introducing publication bias and limiting the generalisability of our findings.*

- **The Results of the quality appraisal of the studies have not been reported.**

This is an important point and we realise that we may have not explained this clearly. The unit of analysis for the quality appraisal in our study is the extracted association, i.e. the individual risk factor-outcome relationship, not the individual article. The quality assessment was performed on each individual association instead of that of the entire article. Hence, one article may have included some risk factor-outcome associations deemed eligible while others were excluded. Similarly, a same article could have included risk factor-outcome associations of low, medium and high quality. More information about our quality appraisal tool has been added to the Methods section on page 11.

The results of the quality appraisal of the risk factor-outcome associations is reported in Figures 3B and 4. We have added a summary of the results of the quality association for each risk factor in the results section. We discuss our quality appraisal further in the discussion (two added paragraphs spanning pp.31-32 on the clean version.) Moreover, all extracted associations, and the associated quality of evidence assessment for each of them is shown in File S3.

*The quality of available evidence differed greatly across the studies and risk factor-outcome associations included in our review. The quality of evidence for taeniasis was particularly poor due to the low sensitivity and lack of species specificity of many tests such as identification of Taenia eggs (159) or self-report of taeniids. More recent tests, such as the rES33 recombinant ELISA, show good sensitivity and specificity without evidence of cross-reactivity (160). Two studies included in our review used this test (17,18). If consistently successful, the rES33 test may improve the quality of evidence for these types of studies by more accurately identifying taeniasis cases and reducing false negatives.*

*The quality of evidence for associations with a porcine cysticercosis outcome also tended to be lower than the quality of evidence for associations with a human cysticercosis outcome. Many of our included studies used lingual exam or meat inspection (see File S3), and neither is a sensitive measure of infection (161). Additionally, since pigs are kept in herds, many of the risk factors related to porcine living conditions would affect the entire group and need to be analyzed through a hierarchical model (162). Our quality assessment graded studies with a combination of two or more serious flaws to be of weak quality (12). While many risk factors for human cysticercosis also act at the group level (e.g. latrine access, free-roaming of pigs), the use of more accurate diagnostic tests for human cysticercosis kept these studies from being graded as having weak quality of evidence.*

- **I believe the discussion section was shallow and lacking enough details to explain some of the important finding reported in this study. For examples the authors should discuss the variation in the strength of evidence as reported by the authors-is it due to issues to do with how the study was conducted, sample size or other contextual issues. A discussion on what was used to gauge the strength of evidence—was it p-values only or were confidence intervals also considered? What created the difference in the number of studies conducted across the different geographical regions. The design of the studies needs to be discussed especially for those focusing on HCC as all this can have an influence on the results reported by the studies.**

Thank you for these excellent points. We separated your comment into four main categories and indicate where we have addressed them in the reviewed manuscript.

- Strength of evidence vs. quality of evidence – Both are referring to the same concept, being the quality appraisal of our risk factor-outcome associations. We have now edited the manuscript, so we solely refer to this appraisal as evaluating the “quality of evidence”.
- Variation in quality of evidence and overall quality of evidence for risk factor-outcome associations (including p-values vs confidence intervals) – this is now discussed in our paragraphs quoted earlier in this letter.
- Difference in number of studies per region –We added a short paragraph in the discussion proposing a reason why the Americas and African regions had relatively more included articles than other WHO regions where TSCT is endemic. We also added a sentence in the paragraph on quality of evidence determination to explain that we used WHO regions to determine whether risk factor-outcome relationships were truly global.

*While our included studies cover all WHO regions, most were from the Americas and African Regions. This may be due to sustained interest and research funding obtained by some research groups over decades. As one example, the Cysticercosis Working Group in Peru has published several studies on the epidemiology of TSCT in Peru (see (23,29,47,83,142,154–156) as a non-exhaustive list.)*

- Study design: We have added this to the second paragraph of our results section. We also discuss study design as a limitation in our methods.

*One limitation of our review is that most identified articles reported on studies applying a cross-sectional design. It can be difficult to infer causality based on such a design, because purported causes and their effects are measured at the same time point, making evaluation of temporality challenging (163). Even with antigen tests that measure active infection, we cannot rule out that modifiable risk factors such as latrine use may have changed between the time of infection and the time of the study (for example, if a program built latrines in the village recently.) This problem of determining temporality is often compounded by the use of antibody-detecting serological tests, which measure prior exposure rather than active infection, thereby further obscuring temporal relationships. Nonetheless, since most individuals with TSCT infections are asymptomatic (85), it remains unlikely that infection itself substantially influenced behavioral patterns in the participants in our included studies.***Addressing Reviewer 2’s Comments**

- **Summary and General Comments and Editorial and Data presentation modifications: The authors conducted a systematic review of the Taenia solium parasite, using the terms “Taenia solium” AND “epidemiology”) They looked for associations between risk factors for taeniasis, human cysticercosis, and porcine cysticercosis. They found a high number of associations, although most of them were weak. Despite this, they found important findings, as reported in lines 331-334.
  However, the authors mention that there are no systematic reviews of TSCT that consider causes of human cysticercosis, human taeniasis, and porcine cysticercosis. Recent reviews summarize the prevalence and geographic distribution of disease in one species, or only consider causation in one species and one region (lines 72-75).
  However, this justification is not entirely correct, as there are some systematic reviews that address this parasitic disease, taking into account human and porcine taeniasis and cysticercosis. An example of this is the publication by Ngwili N et al. (2021) A qualitative assessment of the context and enabling environment for the control of Taenia solium infections in endemic settings. PLoS Negl Trop Dis 15(6): e0009470.** [**https://doi.org/10.1371/journal.pntd.0009470**](https://doi.org/10.1371/journal.pntd.0009470) **In this systematic review, the authors considered the two hosts and three diseases (taeniasis, human cysticercosis, and porcine cysticercosis), and the literature search was global, from 1950 to 2019. Although they do not publicize their work with a One Health approach, Ngwili et al. took into account the same considerations as the present review.
  I believe that this previous work should be taken into account since the present manuscript (Elias Jackson et al.) is not the first systematic review to focus on the three diseases and two hosts involved in the life cycle of Taenia solium.**

Thank you for your comment. It is true that our statement was misleading, and we have now corrected our abstract to state that we are “…the first to consider risk factors of human and porcine *T. solium* disease simultaneously”. We have also made sure to mention that we are interested in “cysticercosis / taeniasis risk factors” and not only cysticercosis / taeniasis. Ngiwili et al’s article reviewed interventions to control *Taenia solium*, and not risk factors or associations.

*Our rigorous systematic review is the first to consider risk factors of human and porcine T. solium disease simultaneously, allowing a synthesis of information from both hosts in order to elucidate causal mechanisms for risk factors of cysticercosis*

**Methods:** Reviewer 2 did not suggest any modifications to the methods section of our manuscript

**Results:** Reviewer 2 made the following comments regarding the results section

- **Figure 1: The life cycle of Taenia solium must be modified. Several freely licensed images can be used without any problem. But if authors are going to make their own drawing of the parasite’s life cycle, they can use different platforms like BioRender, Canva, or other systems to make images more similar to the real thing and make it clear what they’re pointing out. The cysticerci, eggs, Taenia, and pig don’t look alike at all.**

Thank you very much for the program recommendations. We have remade our life cycle image using BioRender and using more cohesive models for hosts and *T. solium* stages. Our use of Biorender is cited in the legend for Figure 1.

- **Figure 2: Figure 2 needs more definition; the writing in the white and blue boxes is barely legible. Although it’s a program-generated image, it could be improved, as it’s not readable at normal size, let alone when enlarged.**

This image was moved into a separate page in landscape mode for larger viewing. Additionally, the final image available online will meet the 300 DPI quality standard set by PLOS NTD, making the image easily enlargeable online.

**Conclusions**: Review 2 felt that we had adequately addressed the different elements of the discussion and conclusion.

**Addressing Your Journal’s Requirements**

- **Please ensure that the PRISMA flowchart describing the included/excluded literature is uploaded as Figure 1.**

We have kept our PRISMA chart as Figure 2 in our manuscript. As confirmed in a follow-up email with the editor, we can keep a figure of the life cycle of *Taenia solium* in the introduction, which makes the PRISMA chart in the Methods Figure 2.
